# Supplementary material for: Functional imaging through scattering medium via fluorescence speckle demixing and localization
Source: ArXiv. 2023 Feb 13:arXiv:2302.06519v1. Preprint. [Version 1] (PMC9949161)
Supplement: Supplement 1 [file NIHPP2302.06519v1-supplement-1.pdf]

Supplementary Information

NMF rank estimation

In order to solve the NMF minimization procedure, the rank of the system needs to be set. This rank is, in an ideal scenario (without noise), the number of sources present in the sample. Given the fact that this number is in principle unknown, a method to estimate it from the experimental data was used. When factorizing the input matrix, I, it is possible to set the rank, k, to any value between 1 and the maximum possible rank of the dataset. Then, it is possible to check the quality of the factorization by looking at the residual error from the NMF ($\|I - W_{est} \cdot H_{est}\|_F^2$). When the rank of the system is underestimated, the factorization procedure tends to merge multiple fingerprints (temporal activities) into single columns (rows) of W_{est} (H_{est}). This implies a high residual error, which becomes smaller as the rank approaches the correct number of sources in the sample. As the rank gets higher than the number of sources, the factorization starts using columns (rows) of W_{est} (H_{est}) to fit the noise present in the measurements, which further reduces the residual error, but at an almost linear rate and with a very small slope. We use this change in the reduction rate of the residual error to estimate the number of sources of the sample. In Fig.5, we show the estimation for the brain slice dataset in the main text with ranks ranging from 1 to 20. In this case, the true number of emitters was 11, which is in good agreement with the region where the slope of the curve changes. Although this method is not exact, we experimentally find that a slight overestimation of the rank neither hinders the capability of the system to retrieve the temporal activities nor the spatial position of the sources. The extra fingerprints recovered when overestimating the rank tend to be high contrast noise-like images, which are discarded by the deconvolution-based localization procedure. In the same manner, the temporal activities from the extra rows of H present random-like signals clearly different from neuronal activity.

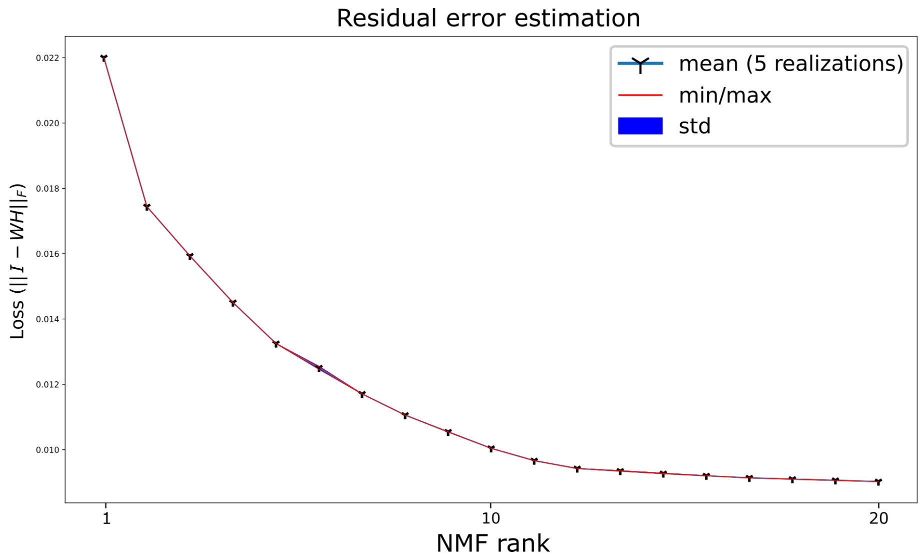

Figure 5: **Rank estimation from experimental data.** For the same recorded dataset, we show the average residual error for five different NMF realizations (with different random initializations) for different rank values. After the rank surpasses the true number of sources in the sample, the residual error decreases at a much lower rate, a phenomena that can be used to estimate the number of emitters in the sample.

NMF inversion problem

In order to solve the general NMF problem, multiple numerical methods can be used[34, 35]. While many of the currently available solvers simply tackle the simplest form of the inversion problem found in the main text ($\min \|I - W \cdot H\|_F^2$ subject to $W, H > 0$), it is possible to add regularization terms with some a priori information about the system, such as the sparsity of either the fingerprints or the temporal activities of the sources in the sample. Thus, we can formulate the NMF problem as:

$$
\begin{aligned}
&\min_{W,H>0} 0.5 \cdot ||I - WH||_\beta^2 \\
&+ \alpha_W \cdot l_{1r} \cdot n_{pixels} \cdot ||vec(W)||_1 \\
&+ \alpha_H \cdot l_{1r} \cdot n_{frames} \cdot ||vec(H)||_1 \\
&+ 0.5 \cdot \alpha_W \cdot (1 - l_{1r}) \cdot n_{pixels} \cdot ||W||_F^2 \\
&+ 0.5 \cdot \alpha_H \cdot (1 - l_{1r}) \cdot n_{frames} \cdot ||H||_F^2,
\end{aligned}
$$

where $||A||_F^2 = \sum_{i,j} A_{ij}^2$ corresponds to the Frobenius norm of a matrix, A, $||vec(A)||_1 = \sum_{i,j} abs(A_{ij})$ corresponds to the l_1 element-wise norm, and $||I - WH||_\beta$ represents the desired β-norm to calculate (1, 2, $\leq$ 0). Here, the additional terms introduced account for both the sparsity of W and H, and are governed by α_W, α_H, and l_{1r} (with two scaling factors, n_{pixels} and n_{frames}, accounting for the vast differences between the number of elements of W and H). Both α_W and α_H can take different values in order to weight the strength of the regularization between W and H, and l_{1r} can be used to continuously choose between different penalty forms. For the limit $l_{1r} = 0$, the penalty behaves like a standard Frobenius norm, while $l_{1r} = 1$ corresponds to an element-wise l_1 penalty (favoring sparsity). In our case, we acquire low contrast images that result of the incoherent addition of many highly-contrasted individual speckles. Furthermore, these patterns do not fully cover the field-of-view of the camera, so some degree of sparsity is to be expected on each individual fingerprint. Moreover, this promotes recoveries where the fingerprints have higher contrast, which greatly helps the localization procedure. Last, the temporal activities that we use to mimic neuronal activity consist of short bursts of activity, usually followed by longer decay times and periods of little activity, so it is reasonable to consider some sparsity on the recovery of H. We find that, in our experimental conditions, a good compromise between fidelity, sparsity, and recovery time is found with $\beta = 2$, $\alpha_W = 1.5$, $\alpha_H = 0.5$, and $l_{1r} = 0.5$. While these regularization parameters have to be manually tuned and are experiment-dependent, several approaches to automatically estimate their values could be explored in the future[36, 37]. The full system is solved by using the *scikit-learn* NMF package[29], and it takes a few minutes to compute for datasets consisting of 500 frames with resolutions in the order of 300×300 pixels using a desktop CPU (Intel i7-9700) with 16 Gb of RAM. Bigger datasets and/or faster reconstruction times could be reached by using GPU-based implementations, but this lies outside of the scope of this work.

Step-by-step localization procedure

Here, we introduce the post-processing workflow to obtain the location of the emitters from the recorded dataset. First, we crop and filter the frames recorded by the camera (as the sensor is larger than the area covered by the speckle patterns). Then, we remove the intensity envelope by high-pass filtering. In the experiments where there is a constant signal present (as in Fig.3), we perform a rank–1 NMF to identify this constant component in the dataset, which we later use to initialize both W and H when performing a full–rank NMF with the rank set to the estimated number of emitters. This helps unmix the time–varying fingerprints and the background present in all the frames due to the constant fluorescence signal. Otherwise, we just initialise the NMF with the Nonnegative Singular Value Decomposition (NNSVD) of the recorded dataset. After the NMF is performed, we deconvolve all the fingerprints in pairs to locate the shifts between them, and finally we merge all the information in the full location map. The codes can be found at [31].

Algorithm 1: Step-by-step localization procedure

Result: Returns the location map of the emitters in the sample by performing NMF over the recorded dataset. The fingerprints provided by the NMF are deconvolved to calculate the distances between the different sources.

Post-process recorded dataset: select the region of the sensor with speckle patterns (cropping), perform binning (reduce size to increase speed) and high-pass filtering (remove envelope, increase contrast)

if *constant background* = *True* **then**
    Do Rank-1 NMF to estimate constant background in the dataset
end

Perform a full–rank NMF on the recorded dataset (n_s = number of sources)
if *constant background* = *True* **then**
    Set $rank = n_s + 1$
    Perform the NMF, initializing W and H with the result of the rank-1 NMF
else
    Set $rank = n_s$
    Perform the NMF, initializing W and H with the Nonnegative Singular Value Decomposition (NNSVD) of the recorded dataset
end

Calculate source positions by deconvolving the fingerprints provided by the NMF algorithm.
for $i = 1 : n_s$ **do**
    for $j = 1 : n_s$ **do**
        Deconvolve $fingerprint_i$ and $fingerprint_j$
        $\delta(x - x_0^{i,j}, y - y_0^{i,j}) = w_i *^{-1} w_j$
        Calculate distance between $source_i$ and $source_j$ as the position of the delta–like peak $(x_0^{i,j}, y_0^{i,j})$
    end
    Combine deconvolutions in a partial location map (M_i):
    $M_i = \sum_{j=1}^{j=n_s} w_i *^{-1} w_j$
end

Correct shifts between all the partial location maps by using the distances between the sources, then add together to generate the full location map
$M = \sum_{i=1}^{i=n_s} M_i(x - x_0^{1,i}, y - y_0^{1,i})$
